# Neuropathologic correlates of cognitive impairment in Alzheimer’s disease with discordant CSF biomarker profiles: co-pathologies in focus

**DOI:** 10.1007/s00401-025-02960-w

**Published:** 2025-11-27

**Authors:** Konstantinos Ioannou, Richard J. Perrin, Khadidzha Abdullaieva, Marina Bluma, Antoine Leuzy, Konstantinos Poulakis, Dorota Religa, Elena Rodriguez-Vieitez, Konstantinos Chiotis

**Affiliations:** 1 Division of Clinical Geriatrics, Center for Alzheimer Research, Department of Neurobiology, Care Sciences and Society, Karolinska Institutet, Neo 7Th Floor 141 52, Huddinge, Stockholm, Sweden; 2 Knight Alzheimer Disease Research Center, Washington University School of Medicine, Saint Louis, USA; 3 Department of Neurology, Washington University School of Medicine, Saint Louis, USA; 4 Department of Pathology and Immunology, Washington University School of Medicine, Saint Louis, USA; 5 Center for Age-Related Diseases, Stavanger University Hospital, Stavanger, Norway; 6 Theme Inflammation and Aging, Karolinska University Hospital, Stockholm, Sweden; 7 Division of Neurogeriatrics, Center for Alzheimer Research, Department of Neurobiology, Care Sciences and Society, Karolinska Institutet, Stockholm, Sweden; 8 Department for Cognitive Disorders, Karolinska University Hospital, Stockholm, Sweden; 9 Department of Neurology, Memory and Aging Center, University of California San Francisco, San Francisco, USA

**Keywords:** Alzheimer, Co-pathologies, ADNC, β-amyloid, p-tau, CSF, AT biomarker system

## Abstract

CSF Aβ reflects Alzheimer’s disease neuropathologic change (ADNC), while CSF p-tau offers an indirect indication of tangle pathology. However, interpretation can be challenging when cognitive impairment is present alongside Aβ positivity (Α +) but p-tau negativity (T −). We examined neuropathologic differences between CSF A + T − and A + T + profiles, defined by CSF Aβ42 and p-tau181 levels, hypothesizing that cognitively impaired older adults with a CSF A + T − profile would exhibit a greater co-pathology burden, suggesting alternative contributing disease processes. We identified 77 ADNI participants with available CSF biomarkers and neuropathologic assessments (median age = 79.8 years; IQR = 74.7–84.5). Depending on the presence–alone or in combination–of ADNC intermediate/high and non-ADNC pathologies (e.g., Lewy bodies (LB), argyrophilic grain disease (AGD), limbic-predominant age-related TDP-43 encephalopathy-neuropathologic change (LATE-NC)), individuals were classified as ADNC dominant, mixed ADNC, or non-ADNC dominant. The two CSF A + profiles were similar in demographics, frequency of cognitive impairment, longitudinal cognitive performance, clinical comorbidities, CSF Aβ42 levels, CSF α-synuclein positivity rates, and Aβ PET burden. ADNC intermediate/high was significantly more frequent in the CSF A + T + profile than in the CSF A + T − profile (100% vs. 78%, *p* value = 0.008). The most common co-pathologies contributing to cognitive impairment in the CSF A + T − profile were LATE-NC (stages 2–3) (47%), LB limbic/neocortical (44%), and AGD (stages II–III) (33%), while in the CSF A + T + profile, LB limbic/neocortical (28%) and LATE-NC (stages 2–3) (22%) predominated. The CSF A + T − profile showed 17% ADNC dominant, 61% mixed ADNC, and 22% non-ADNC dominant pathology, whereas the CSF A + T + profile showed 51% ADNC dominant and 49% mixed ADNC pathology (*p* = 0.001). Within the mixed ADNC subgroup, individuals with a CSF A + T − profile more often exhibited two or more non-ADNC co-pathologies compared to those with a CSF A + T + profile (73% vs. 21%, *p* = 0.009). Despite clinical similarities among cognitively impaired individuals with CSF A + T − and A + T + profiles, the CSF A + T − profile may reflect a greater burden of non-ADNC pathology. Extending biomarker profiling beyond Aβ and tau may facilitate more personalized care.

**Supplementary Information:**

The online version contains supplementary material available at 10.1007/s00401-025-02960-w.

## Introduction

Positivity for an established β-amyloid (Aβ) biomarker (A +) indicates that an individual likely has Alzheimer’s disease neuropathologic change (ADNC), but Aβ status alone is not sufficient to establish the etiology of cognitive decline [42]. The increasing prevalence of Aβ pathology with aging further challenges the interpretation of A + as sufficient evidence that cognitive decline is solely attributable to ADNC [27, 56]. Indeed, among A + individuals, the extent of tau pathology can vary substantially and, in the setting of ADNC, tau burden exhibits a stronger correlation with cognitive impairment than Aβ alone [7, 9]. The use of cerebrospinal fluid (CSF) Aβ and tau biomarkers in memory clinics has become widely adopted. Nevertheless, the scarcity of CSF–pathology studies with detailed postmortem characterization–beyond the presence or absence of AD pathologic features–hinders a comprehensive understanding of the clinical interpretation of these biomarkers with respect to the underlying neuropathologic mechanisms.

Until recently, the clinical relevance of mixed pathologies (i.e., the presence of co-pathologies) in the diagnosis and prognosis of cognitive impairment had been underestimated, although their prevalence may exceed 50% in the elderly [54, 57, 70]. Interestingly, in cases of mixed pathologies, the total pathologic burden, rather than individual pathologies, best explains the severity of cognitive impairment [29, 50, 58]. Proteinopathies associated with α-synuclein (e.g., Lewy bodies (LB) spectrum disorders), TDP-43 (e.g., frontotemporal lobar degeneration (FTLD-TDP43), limbic-predominant age-related TDP-43 encephalopathy-neuropathologic change (LATE-NC)), [29, 30, 45, 46] non-AD tau (e.g., FTLD-tau, argyrophilic grain disease (AGD)), [58] as well as vascular brain injury (VBI) [3] commonly coexist with ADNC as postmortem findings [12, 31, 33]. Despite the potential contribution of these pathologies to cognitive impairment, the frequency and clinical relevance of co-existing ADNC and non-ADNC pathologies have not been rigorously investigated in the context of CSF Aβ and tau biomarkers, particularly among CSF A + individuals with a low phosphorylated tau burden (i.e., discordant CSF A + T −) compared to CSF A + cases with a high tau burden (i.e., concordant CSF A + T +).

We aimed to investigate the neuropathologic differences between cognitively impaired older adults with discordant and concordant CSF A/T biomarker profiles (i.e., A + T − vs. A + T +) focusing on the co-existence of ADNC and non-ADNC pathologies. Addressing this question is an essential first step toward evaluating the impact of co-pathologies on interpreting the cause of cognitive impairment within the framework of CSF A/T biomarker profile system. To this end, we identified participants from the Alzheimer’s Disease Neuroimaging Initiative (ADNI) with available in vivo biomarker profiling, longitudinal cognitive assessments, comprehensive medical histories and standardized postmortem neuropathologic assessments. After grouping individuals according to their CSF A/T biomarker profiles, we evaluated (1) their clinical characteristics and (2) the frequencies of ADNC, non-ADNC pathologies associated with cognitive impairment, as well as their co-occurrence (mixed ADNC). The overall objective was to investigate whether the presence and the number of co-pathologies differ in CSF A + individuals with varying phosphorylated tau levels (i.e., A + T − vs. A + T +). Our hypothesis is that, in CSF A + T − individuals, cognitive impairment may be more frequently attributable to both ADNC and non-ADNC pathologies, reflecting the earlier stage of AD-related tau pathology. In contrast, among CSF A + T + individuals, ADNC is more likely to be the dominant underlying pathology.

## Methods

### Participants

Participants were selected from the ADNI database (Supplementary Table 1). ADNI was launched in 2003 and is a multicenter study conducted as a public–private partnership under the leadership of Principal Investigator Michael W. Weiner, MD. ADNI aims to test the feasibility of combining imaging techniques, biofluid markers, and clinical and neuropsychological assessment for monitoring mild cognitive impairment (MCI) and early AD.

We identified 77 ADNI participants with available postmortem neuropathologic assessments who had provided at least one antemortem CSF sample. For participants who had provided multiple CSF samples, data from the most recent sample (i.e., the one with the minimal time difference from the autopsy) were used to define the individual’s biomarker status. Individuals were classified according to their CSF A/T status into four groups: CSF A − T − , A − T + , A + T − , and A + T + [25]. Based on the clinical diagnosis at the time of biomarker testing, the individuals were also grouped into cognitively unimpaired and impaired (i.e., individuals diagnosed with MCI or dementia) [41].

### Biomarkers

#### CSF biomarkers

CSF Aβ_1-42_ (Aβ42) and CSF total and phosphorylated tau (p-tau181) levels were measured with the fully automated Roche Elecsys immunoassay to define CSF A/T status at the University of Pennsylvania [4, 5]. CSF A + was defined as CSF Aβ42 ≤ 981 pg/mL, while CSF T + was defined as CSF p-tau181 ≥ 24.3 pg/mL. Both these cut-offs have been previously validated in a similar population against PET imaging cut-offs [16]. CSF α-synuclein (α-syn) aggregation was measured using the synuclein seed amplification assay at the Amprion Clinical Laboratory [63].

#### Aβ PET imaging

A subset of the study participants (*n* = 51) underwent one or more Aβ positron emission tomography (PET) scans with ^11^C-PIB, ^18^F-Florbetapir, or ^18^F-Florbetaben, as previously described [36, 43]. Cortical uptake was quantified, in-house, relative to the uptake in the whole cerebellum (reference region) using the standard Centiloid pipeline and the respective equations for scaling standard uptake value ratios to Centiloids (CL) [32, 44, 60]. Aβ PET positivity was defined based on a cut-off value of > 24.4 CL [28].

### Cognitive measures

Longitudinal Mini-Mental State Examination (MMSE) and ADNI composite memory (ADNI-Mem) scores were used to assess cognitive performance [10].

### Clinical comorbidities and medication

Two medically trained investigators (KA, KI) assessed participants’ medical histories (Supplementary Table 1), for clinical comorbidities with a potential effect on cognition, as previously described [24]. The vascular risk factors burden, [20] and the anticholinergic medication burden (ACB), arising from commonly prescribed medications, were also evaluated [21, 37].

### Neuropathology

All neuropathologic assessments were performed by the ADNI Neuropathology Core (NPC) at Washington University (Supplementary Table 1). The format of the Neuropathology Data Form Version 10 or 11 of the National Alzheimer’s Coordinating Center (NACC) established by the National Institute on Aging/National Institutes of Health (NIA/NIH, U01 AG016976) was used by the ADNI NPC to catalog the neuropathologic findings for each case [42]. Neuropathologies with potential relevance to cognitive impairment included Aβ plaques, neurofibrillary tangles (NFT), other non-AD tau pathologies, TDP-43 pathology, LB, hippocampal sclerosis (HS), and VBI [52]. Subsequently, established staging schemes were applied to each pathology to determine both the individual neuropathologic diagnosis and its corresponding stage. Each diagnosis was then binarized based on standard criteria to indicate whether the burden of a given pathology was considered sufficient, in isolation, to plausibly contribute substantially to cognitive impairment (Table 1, Supplementary Methods; Neuropathology). As an example, TDP-43 pathology—when not consistent with FTLD-TDP43—was staged according to the LATE-NC classification scheme. LATE-NC stages 2–3 were considered sufficient to plausibly contribute to cognitive impairment in isolation, while stage 1, limited to the amygdala, was not considered an adequate contributor on its own [45, 46]. Similar schemes were applied to the other neuropathologies [1, 2, 8, 11, 13–15, 22, 34, 38–40, 52, 61, 66]. The composite neuropathologic profile for each individual was determined, classifying them as ADNC dominant (if only ADNC intermediate/high was deemed capable of contributing to impairment according to our prespecified criteria), mixed ADNC (if, in addition to ADNC intermediate/high, other non-ADNC pathologies were identified as potential contributors to impairment), or non-ADNC dominant (if only non-ADNC pathologies were identified). In cases of mixed ADNC the number of co-pathologies was recorded. VBI was not included in our definition of mixed ADNC, as its contribution to cognitive impairment likely depends on factors not available in our dataset, such as the exact location of the lesions. A sensitivity analysis of the composite neuropathologic profile, including VBI within non-ADNC pathologies and in the definition of mixed ADNC, is presented in the Supplementary Material.

**Table 1:** Binary classification of neuropathologies based on their plausible contribution to cognitive impairment

Pathologies potentially associated with cognitive impairment	Threshold for relevance in this study (based on NACC guidelines version 10 or 11)^1^	Key references
Alzheimer’s disease neuropathologic change (ADNC)	Intermediate or high vs. none or low	Montine TJ, et al., 2012
Lewy bodies (LB)	Neocortical or limbic vs. none or amygdala or brainstem or olfactory bulb^**2**^	McKeith IG, et al., 2005 McKeith IG, et al., 2017
Progressive supranuclear palsy (PSP)	Presence vs. absence	Kovacs GG, et al., 2020
Argyrophilic grain disease (AGD)^**3**^	Stage II–III vs. none or Stage I	Saito Y, et al., 2004 Tolnay M, et al. 2004
Frontotemporal lobar degeneration (TDP-43) (FTLD)	Presence vs. absence	Cairns NJ, et al., 2007 Mackenzie IRA, et al., 2011
Limbic-predominant age-related TDP-43 encephalopathy—neuropathologic change (LATE-NC)^**4**^	Stage 2–3 vs. none or Stage 1	Montine TJ, et al., 2012 Nelson PT, et al., 2019, 2023
Hippocampal sclerosis (HS)	Presence (at least unilateral) vs. absence	Amador-Ortiz C, et al., 2008 Montine TJ, et al., 2012
Primary age-related tauopathy (PART)	Definitive or possible PART vs. absence	Crary JF, et al., 2014
Vascular brain injury (VBI) [any of these]^**5**^	Old infarcts observed grossly, including lacunes Old microinfarcts (not observed grossly) Single/multiple old hemorrhages observed grossly Old cerebral microbleeds (≤ 5 mm)	Montine TJ, et al., 2012

^1^ Neuropathology data were catalogued in the format of the neuropathology data form version 10 or 11 of the NACC established by the National Institute on Aging/NIH (U01 AG016976). ^2^ Predominant presence of Lewy bodies in any of these regions. ^3^ Because the NACC form (and therefore the ADNI data currently available from LONI) does not include AGD staging, this information was obtained after communication with the ADNI NPC. All other forms of FTLD-tau (e.g., PSP, CBD) were assessed in a dichotomous manner (presence vs. absence). ^4^ Because the NACC form (and therefore the ADNI data currently available from LONI) does not readily support the original or updated staging systems for LATE-NC (Nelson PT, et al., 2019, 2023), the classification of cases used in this article was obtained after communication with the ADNI NPC and a detailed review of cases identified as having TDP-43 pathology. ^5^ Standard screening sections described in Perrin RJ, et al., 2024. *ADNI NPC* Alzheimer’s Disease Neuroimaging Initiative Neuropathology Core, *CBD* corticobasal degeneration, *LONI* laboratory of neuro imaging, *NACC* National Alzheimer’s Coordinating Center

### Statistical analysis

Statistical analysis was carried out in R (4.1.1) with standard R libraries. The CSF A/T groups were compared in terms of demographics, biomarker levels, cognitive performance, frequency of clinical comorbidities, and neuropathologies. Kruskal–Wallis and Wilcoxon pairwise tests were used for group comparisons in case of continuous data, whereas Fisher’s exact test was used for categorical data. In addition, the association between CSF A/T group and the number of co-pathologies in cases with mixed ADNC was also examined. A modified version of the typical Venn diagrams was used to visualize the intersections between the neuropathologic findings across the CSF A/T groups [51]. The performance of CSF Aβ42 and p-tau181 in detecting AD pathologic features, and of CSF α-syn in detecting LB pathology was also evaluated.

Longitudinal cognitive performance was assessed using separate linear mixed-effects models for MMSE and ADNI-Mem, allowing for comparison of the rate of cognitive decline across the CSF A/T groups (Supplementary Methods; Longitudinal cognitive modelling). *α* = 0.05 was set as the level of statistical significance.

### Standard protocol approvals, registrations, and patient consents

ADNI has been carried out in strict adherence to Good Clinical Practice guidelines and Regulations for the Protection of Human Subjects in Research. It aligns with Institutional Review Boards (IRB), International Conference on Harmonization, Health Insurance Portability and Accountability Act, State and Federal regulations and all other local regulatory requirements and laws. All individuals provided signed IRB-approved written informed consent forms.

An additional ethical approval was obtained from the regional Human Ethics Committee of Stockholm (Etikprövningsmyndigheten) for the analysis of the ADNI data (Dnr 2024–08722-01).

## Results

### Participants

We identified 77 ADNI participants who had available CSF and neuropathologic data and grouped them based on their positivity for CSF Aβ42 and p-tau181 into CSF A/T groups. The median [interquartile range, IQR] interval between CSF biomarker testing and death was 3.1 [1.5, 5.7] years. This interval was similar across the CSF A/T groups (Table 2).

**Table 2:** Demographics, clinical information, and biomarker levels across the CSF A/T groups

Characteristic	A−T−, *N* = 10^1^	A−T + , *N* = 10^1^	A + T−, *N* = 18^1^	A + T + , *N* = 39^1^	*p* value^2^	
Demographics and APOE					All A/T groups	A + T− vs A + T +
Age at CSF sampling (y.)	77.5 (72.6, 86.0)	83.0 (80.3, 84.7)	80.5 (76.2, 85.0)	76.3 (73.0, 82.0)	0.2	
Sex (Female)	0/10 (0%)	5/10 (50%)	3/18 (17%)	12/39 (31%)	**0.042**	NS
Education (y.)	17.5 (15.3, 18.0)	17.0 (14.3, 18.0)	18.0 (16.0, 19.8)	16.0 (15.0, 18.0)	0.5	
APOE ε4 carriers	1/10 (10%)	3 / 10 (30%)	4/18 (22%)	31 / 39 (79%)	** < 0.001**	** < 0.001**
Cognitive status						
Cognitive impairment						
At CSF sampling	5/10 (50%)	7/10 (70%)	17/18 (94%)	38/39 (97%)	** < 0.001**	NS
Last assessment	6/10 (60%)	8/10 (80%)	17/18 (94%)	39/39 (100%)	**0.001**	NS
Neuropsychology^3^						
MMSE	29.0 (28.3, 30.0)	27.0 (26.3, 28.0)	23.0 (20.5, 26.8)	24.0 (19.5, 26.0)	** < 0.001**^**4**^	NS
ADNI-Mem^**5**^	0.2 (0.0, 1.3)	−0.1 (−0.6, 1.1)	−0.7 (−1.3, 0.4)	−1.1 (−1.5, −0.7)	** < 0.001**^**6**^	NS
Biomarkers						
Interval from CSF sampling to death (y.)	3.4 (1.9, 6.4)	1.6 (0.7, 4.7)	2.8 (1.3, 5.7)	3.8 (1.0, 5.2)	0.5	
CSF Aβ42 (pg/mL)	1391.5 (1230.3, 1605.8)	2016.0 (1061.0, 3052.3)	480.4 (358.9, 570.6)	540.2 (421.1, 665.9)	** < 0.001**	NS
CSF total tau (pg/mL)	195.4 (169.3, 215.8)	416.7 (339.4, 461.1)	215.2 (193.0, 232.1)^**7**^	361.5 (296.3, 450.8)	** < 0.001**	** < 0.001**
CSF p-tau181 (pg/mL)	15.6 (13.6, 17.6)	32.8 (30.0, 39.7)	20.4 (17.2, 23.1)^**7**^	34.3 (28.6, 46.3)	** < 0.001**	** < 0.001**
CSF α-syn^**8,9**^ (Type 1) +	2/9 (22%)	4/10 (40%)	9/18 (50%)	15/38 (39%)^**10**^	0.8	
Not assessed	1	0	0	0		
Aβ PET burden (CL)^**11**^	−2.0 (−9.4, 4.8)	−6.8 (−14.6, 61.9)	72.8 (44.8, 85.4)	94.1 (66.8, 113.8)	** < 0.001**	NS
Not assessed	3	4	8	11		

The bold represent the significant statistical comparisons

^1^ Median (interquartile range); *n*/*N* (%). ^2^ Kruskal–Wallis rank sum test; Fisher’s exact test. ^3^ At the time of CSF sampling. ^4^ Both the CSF A + T− and A + T + groups showed significantly lower MMSE scores than the CSF A−T− group (*p* = 0.002 and *p* < 0.001, respectively). ^5^ ADNI composite memory score. ^6^ Both the CSF A + T− and A + T + groups showed significantly lower ADNI-Mem scores than the CSF A−T− group (*p* = 0.039 and *p* < 0.001, respectively). ^7^ One CSF A + T− individual was excluded from this statistical analysis due to exceptionally low CSF total tau and CSF p-tau181 levels. ^8^ Positivity Type 1=αSyn aggregates detected, aggregation profile consistent with Type 1 seeds as seen in Parkinson’s disease and Lewy body dementia; Positivity Type 2=αSyn aggregates detected, aggregation profile consistent with Type 2 seeds as seen in multiple system atrophy. ^9^ No individual showed Type 2 positivity. ^10^ The result for one CSF A + T + individual was characterized as indeterminate and was not included in the analysis. ^11^ The interval between CSF sampling and Aβ PET did not differ across the CSF A/T groups (all individuals underwent Aβ PET within the same year as the CSF sampling, except for two CSF A−T− individuals who underwent Aβ PET 2.9 and 5 years after CSF sampling, and one CSF A + T + individual who underwent Aβ PET 1.8 years before CSF sampling). *A − /* + β-amyloid negativity/positivity, *ADNI* Alzheimer’s Disease Neuroimaging Initiative, *CSF* cerebrospinal fluid, *MMSE* Mini-Mental State Examination, *PET* positron emission tomography, *T − /* + tau negativity/positivity

### Demographics and frequency of APOE ε4

There were no demographic differences between the two CSF A + groups (i.e., A + T − vs. A + T +). The frequency of APOE ε4 carriership was significantly higher in CSF A + T + individuals than in CSF A + T − individuals (Table 2).

### Frequency of cognitive impairment and longitudinal cognitive performance

At the time of biomarker testing, the CSF A + T − and A + T + groups were significantly more impaired in MMSE and ADNI-Mem compared to the CSF A − T − group. No significant differences were observed between the two CSF A + groups in the frequency of cognitively impaired cases, or in MMSE and ADNI-Mem scores (Table 2). Premortem, 94% of CSF A + T − and 100% of CSF A + T + individuals were cognitively impaired.

**Fig. 1:**
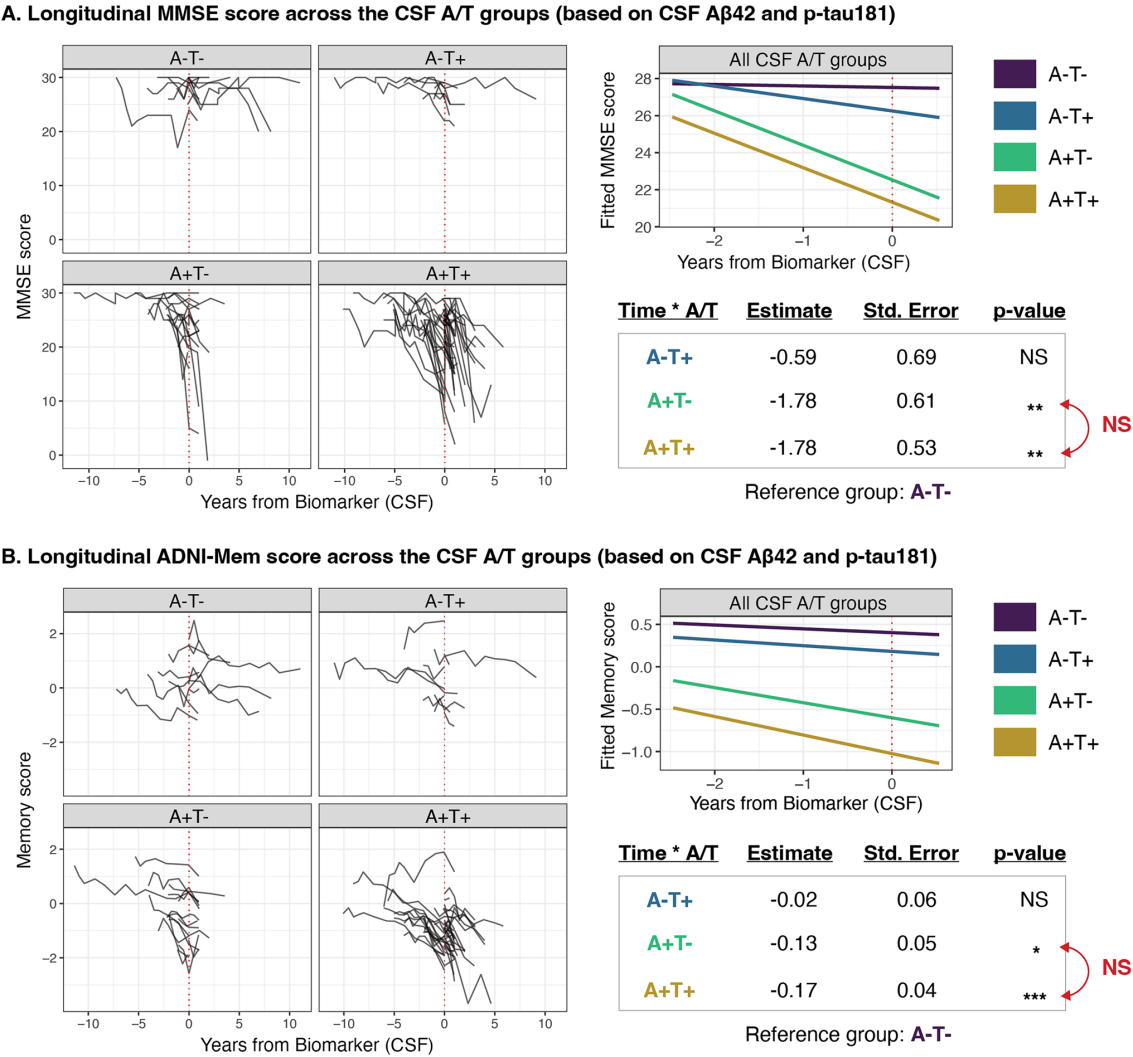
Longitudinal neuropsychological assessment across the CSF A/T groups (based on CSF Aβ42 and p-tau181). **A** Spaghetti plots representing all the MMSE scores for each individual (left) and longitudinal modeling of cognitive performance based on MMSE across the CSF A/T groups using LME models (right). **B** Spaghetti plots representing all the ADNI-Mem (ADNI composite memory) scores for each individual (left) and longitudinal modeling of cognitive performance based on ADNI-Mem across the CSF A/T groups using LME models (right). The time point zero represents the time of CSF sampling. One CSF A + T− individual was excluded from longitudinal modeling due to a lack of longitudinal data (i.e., this individual had only one neuropsychological assessment available for both the MMSE and the ADNI composite memory score). *A − /* + β-amyloid negativity/positivity, *ADNI* Alzheimer’s Disease Neuroimaging Initiative, *CSF* cerebrospinal fluid, *LME* linear mixed-effects models, *MMSE* Mini-Mental State Examination, *NS* no statistically significant difference, *T − /* + tau negativity/positivity]

Longitudinally, the interaction term of CSF A/T status and time was significant in both models concerning MMSE and ADNI-Mem. The rate of decline was significantly steeper in both the CSF A + T- and A + T + groups, compared to the CSF A − T − group, for both MMSE and ADNI-Mem. However, no significant differences in the rate of cognitive decline were observed between the two CSF A + groups or between the two CSF A- groups for either cognitive measure (Fig. 1). The longitudinal changes in clinical diagnosis and cognitive status for all individuals are shown in Supplementary Figs. 1, 2.

### CSF biomarkers and Aβ PET

We evaluated CSF Aβ42 and p-tau181 levels across Thal Aβ phases, Braak NFT stages, CERAD (i.e., consortium to establish a registry for Alzheimer’s disease) scores and ADNC classifications, and examined their performance in detecting binary-defined pathologic thresholds (Supplementary Fig. 3, Supplementary Table 2). The performance of CSF α-syn biomarker for detecting LB pathology is shown in Supplementary Table 2. CSF Aβ42 levels were similar between the CSF A + T − and A + T + groups, as was the Aβ PET burden (Table 2). Four individuals showed discrepancy between CSF Aβ42 status and Aβ PET status (Supplementary Table 3, Supplementary Fig. 4). The longitudinal biomarker status for all individuals is reported in Supplementary Fig. 5. No significant differences in proportions of CSF α-synuclein positivity were observed across the CSF A/T groups (Table 2).

### Clinical comorbidities and medication

Except for hypertension, which was significantly more frequent in the CSF A + T + group than in the CSF A + T − group, the frequency of other clinical comorbidities was similar between the two CSF A + groups, as were the rates of AD medication prescriptions and ACB + (Supplementary Table 4).

### Composite Neuropathologic profile and non-ADNC neuropathologic burden

**Fig. 2:**
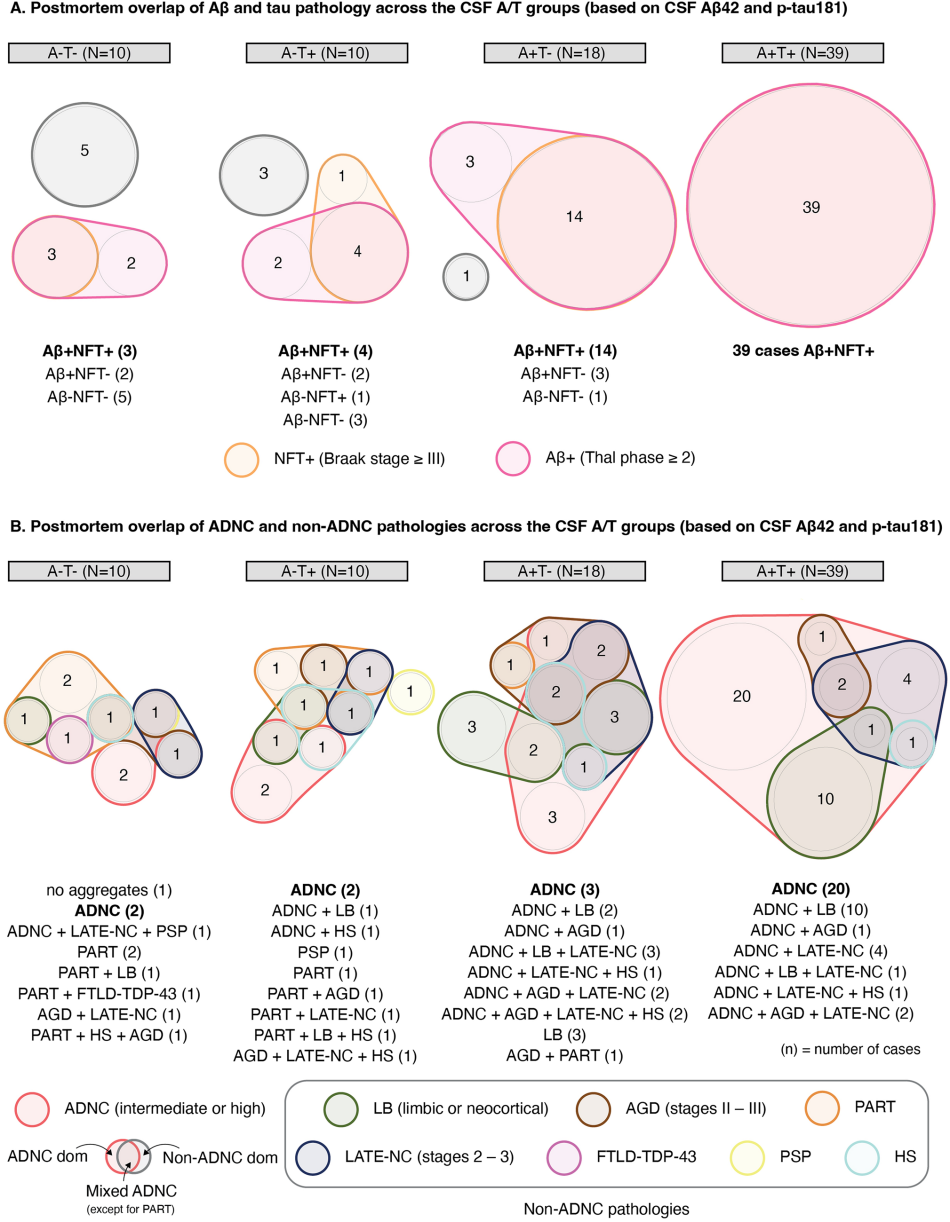
Overlapping pathologies across the CSF A/T groups (based on CSF Aβ42 and p-tau181). **A** Postmortem overlap of Aβ and tau pathology by CSF A/T status. **B** Postmortem overlap of ADNC and other non-ADNC pathologies by CSF A/T status. The postmortem findings of each individual in all CSF A/T groups are shown. ADNC indicates the likelihood of neuropathologic evidence of AD. ADNC intermediate/high is considered a sufficient explanation for dementia. Four individuals (A−T + ; *n* = 1, A + T−; *n* = 1, A + T + ; *n* = 2) have not been assessed regarding the presence of LATE-NC (Table 3). Composite neuropathologic profiles were defined as follows: ADNC dominant = only ADNC intermediate/high was present. Mixed ADNC = ADNC intermediate/high co-occured with ≥ 1 non-ADNC pathologies (PART is not included in mixed ADNC by default). Non-ADNC dominant = any of the seven non-ADNC pathologies was present in the absence of ADΝC intermediate/high. *A − /* + β-amyloid negativity/positivity, *AD* Alzheimer’s disease, *ADNC* Alzheimer’s disease neuropathologic change, *AGD* Argyrophilic grain disease, *CSF* cerebrospinal fluid, *FTLD* frontotemporal lobar degeneration, *HS* hippocampal sclerosis, *LATE-NC* limbic-predominant age-related *TDP-43* encephalopathy neuropathologic change, *LB* Lewy bodies, *NFT* neurofibrillary tangles, *PART* primary age-related tauopathy, *PSP* progressive supranuclear palsy, *T − /* + tau negativity/positivity]

Table 3 presents the results of the neuropathology analyses across the CSF A/T groups. The frequency of Aβ pathology Thal phase ≥ 2 showed no significant difference between the two CSF A + groups. NFT pathology at Braak stages ≥ III and ≥ V was significantly more frequent in the CSF A + T + group than in the CSF A + T − group. The frequency of ADNC intermediate/high increased stepwise across the CSF A/T groups and differed significantly between the CSF A + T − and A + T + groups. Compared to the CSF A + T + group, the CSF A + T− group had a higher frequency of different non-ADNC pathologies (i.e., LATE-NC (stages 2–3), LB limbic/neocortical, AGD (stages II–III), and HS), but only the frequency of AGD stages II–III reached statistical significance. For a more detailed breakdown of the cases with LB, [39, 40] see Supplementary Table 5. No cases showed evidence of amyotrophic lateral sclerosis, Pick’s disease or corticobasal degeneration. The composite neuropathologic profiles differed significantly between the CSF A + T − and A + T + groups, with the CSF A + T − group exhibiting a higher frequency of non-ADNC dominant or mixed ADNC pathology compared to the CSF A + T + group, which more often exhibited an ADNC dominant pathology. Among cases with mixed ADNC, the frequency of individuals with two or more non-ADNC co-pathologies was significantly higher in the CSF A + T − group compared to the CSF A + T + group. In the CSF A − T + group, the most common composite neuropathologic profile was non-ADNC dominant, though mixed-ADNC and ADNC dominant cases were also observed. Figure 2A illustrates a breakdown of the overlap between Aβ pathology (Thal phase ≥ 2) and NFT pathology (Braak NFT stage ≥ III) across the CSF A/T groups, while Fig. 2B illustrates the overlap of all neuropathologic diagnoses of interest across the CSF A/T groups. Supplementary Table 6 presents the results of the neuropathology analyses when VBI was included as a component of the composite neuropathologic profile.

**Table 3:** Postmortem findings across the CSF A/T groups

Pathology	A−T− (*N* = 10)^1^	A−T + (*N* = 10)^1^	A + T− (*N* = 18)^1^	A + T + (*N* = 39)^1^	*p* value^2^	
Pathology related to AD					All A/T groups	A + T- vs A + T +
Aβ plaques, Thal phase ≥ 2	5/10 (50%)	6/10 (60%)	17/18 (94%)	39/39 (100%)	** < 0.001**	NS
NFT, Braak stage ≥ III	3/10 (30%)	5/10 (50%)	14/18 (78%)	39/39 (100%)	** < 0.001**	**0.008**
NFT, Braak stage ≥ V	0/10 (0%)	5/10 (50%)	9/18 (50%)	38/39 (97%)	** < 0.001**	** < 0.001**
Neuritic plaques (CERAD score)^**3**^	1/10 (10%)	4/10 (40%)	11/18 (61%)	38/39 (97%)	** < 0.001**	** < 0.001**
ADNC (intermediate/high)^**4**^	3/10 (30%)	4/10 (40%)	14/18 (78%)	39/39 (100%)	** < 0.001**	**0.008**
Non-ADNC pathologies that, in isolation, have been associated with cognitive impairment						
LB (limbic/neocortical)	1/10 (10%)	2/10 (20%)	8/18 (44%)	11/39 (28%)	0.3	
PSP	1/10 (10%)	1/10 (10%)	0/18 (0%)	0/39 (0%)	0.065	
AGD (stages II–III)^**5**^	2/10 (20%)	2/10 (20%)	6/18 (33%)	3/39 (8%)	0.082	**0.022**
FTLD (TDP-43)	1/10 (10%)	0/10 (0%)	0/18 (0%)	0/39 (0%)	0.3	
LATE-NC (stages 2–3)^**6**^	2/10 (20%)	2/9 (22%)	8/17 (47%)	8/37 (22%)	0.3	
Not assessed	0	1	1	2		
HS^**7**^	1/10 (10%)	3/10 (30%)	3/18 (17%)	1/39 (3%)	**0.032**	NS
PART	5/10 (50%)	4/10 (40%)	1/18 (6%)	0/39 (0%)	** < 0.001**	NS
Composite neuropathologic profile					** < 0.001**	**0.001**
ADNC dominant^**8**^	2/10 (20%)	2/10 (20%)	3/18 (17%)	20/39 (51%)		
Mixed ADNC^**9,10**^	1/10 (10%)	2/10 (20%)	11/18 (61%)	19/39 (49%)		
Non-ADNC dominant^**11**^	6/10 (60%)^**12**^	6/10 (60%)	4/18 (22%)	0/39 (0%)		
Number of non-ADNC co-pathologies in mixed ADNC cases					**0.005**	**0.009**
ADNC + 1 non-ADNC co-pathology	0/1 (0%)	2/2 (100%)	3/11 (27%)	15/19 (79%)		
ADNC + ≥ 2 non- ADNC co-pathologies	1/1 (100%)	0/2 (0%)	8/11 (73%)	4/19 (21%)		
VBI	3/10 (30%)	2/10 (20%)	7/18 (39%)	9/38 (24%)	0.7	
Not assessed	0	0	0	1		
Pathologies that, in isolation, have not been associated with cognitive impairment						
CAA (moderate/severe)	0/10 (0%)	1/10 (10%)	7/18 (39%)	20/39 (51%)	**0.003**	NS
Neuronal loss in SN^**13**^	1/8 (12%)	0/9 (0%)	6/18 (33%)	4/38 (11%)	0.084	
Not assessed	2	1	0	1		

The bold represent the significant statistical comparisons

^1^ *n*/*N* (%). ^2^ Fisher’s exact test. ^3^ Moderate or frequent density of neuritic plaques. ^4^ According to NACC guidelines, evidence o ADNC was classified as absent, low, intermediate, or high based on combined information from Thal Aβ phases, Braak NFT staging, and CERAD score for density of neuritic plaques. ADNC intermediate/high is considered a sufficient explanation for dementia. ^5^ Because the NACC form (and therefore the ADNI data currently available from LONI) does not include AGD staging, this information was obtained after communication with the ADNI NPC. ^6^ Because the NACC form (and therefore the ADNI data currently available from LONI) does not readily support the original or updated staging systems for LATE-NC (Nelson PT, et al., 2023), the classification of cases used in this article was obtained after communication with the ADNI NPC and a detailed review of cases identified as having TDP-43 pathology. ^7^ Severe neuronal loss and gliosis in CA1 and/or subiculum, at least unilateral, unexplained by NFT pathology. ^8^ ADNC dominant = if only ADNC intermediate/high was present. ^9^ Mixed ADNC = ADNC intermediate/high +  ≥ 1 non-ADNC pathologies that, in isolation, have been associated with cognitive impairment. ^10^ PART is not included in the definition of mixed ADNC by default. ^11^ Non-ADNC dominant = presence of any of the seven non-ADNC pathologies in the absence of ADΝC intermediate/high. ^12^ One CSF A−T− individual (10%) had neither ADNC nor any of the seven non-ADNC pathologies, each of which has been independently associated with cognitive impairment, examined in this study. ^13^ Moderate or severe loss. *A − /* + β-amyloid negativity/positivity, *ADNC* AD neuropathologic change, *ADNI NPC* Alzheimer’s Disease Neuroimaging Initiative Neuropathology Core, *AGD* argyrophilic grain disease, *CAA* cerebral amyloid angiopathy, *CERAD* consortium to establish a registry for Alzheimer’s disease, *CSF* cerebrospinal fluid, *FTLD* frontotemporal lobar degeneration, *HS* hippocampal sclerosis, *LATE-NC* limbic-predominant age-related *TDP-43* encephalopathy neuropathologic change, *LB* Lewy bodies, *LONI* laboratory of neuro imaging, *NACC* National Alzheimer’s Coordinating Center, *NFT* neurofibrillary tangles, *PART* primary age-related tauopathy, *PSP* progressive supranuclear palsy, *SN* substantia nigra, *T − /* + tau negativity/positivity, *VBI* vascular brain injury]

## Discussion

In cases with positive core AD biomarkers, the prevalence of non-ADNC pathologies at postmortem neuropathologic assessment is high, suggesting their potentially complex role in clinically relevant cognitive impairment. Our analyses revealed that: 1.) although the individuals with CSF A + profiles, irrespective of T status (i.e., A + T − and A + T +), exhibited essentially indistinguishable clinical phenotypes, there were neuropathologic differences between the CSF A + T − and A + T + groups, 2.) the frequency of ADNC intermediate/high was highest in the CSF A + T + group, and 3.) the CSF A + T − group, compared to the CSF A + T + group, exhibited a greater burden of non-ADNC neuropathologies, both in terms of composite profiles and co-pathology load in cases with mixed ADNC.

Aβ biomarker positivity is considered a defining diagnostic feature of AD, although Aβ pathology frequently coexists with other non-ADNC pathologies [18, 19, 48]. In cases of cognitive impairment, where a positive Aβ biomarker is accompanied by a negative tau biomarker, the clinical interpretation with respect to underlying pathology may be more complex [24]. We conducted a CSF—pathology study to examine differences in the frequency of co-occurring ADNC and non-ADNC pathologies between CSF A + T − and A + T + cognitively impaired older adults. In our study, the CSF A + T − and A + T + groups had similar clinical characteristics. They showed similar rates of cognitive decline, had comparable demographics, clinical comorbidities, Aβ PET burden and CSF α-synuclein positivity rates, and were prescribed AD medication at similar frequencies. This phenotypic similarity suggests that, in the absence of tau biomarker information, CSF A + T − and A + T + individuals may appear clinically similar, and their cognitive impairment could be interpreted as stemming from a shared underlying pathology—namely ADNC [69]. However, given that tau biomarkers reflect, to some extent, the underlying NFT burden, it is plausible that CSF A + T − individuals are at an earlier stage of tau pathology. Our hypothesis was that neuropathologic differences may exist between the CSF A + T − and A + T + groups. According to our hypothesis, in CSF A + T− individuals, a greater co-occurrence of ADNC and non-ADNC pathologies [6] that can contribute to cognitive impairment might effectively advance their clinical stage beyond what would be expected based solely on their biological AD stage. We investigated this hypothesis within the framework of the recently revised AD criteria [26].

**Fig. 3:**
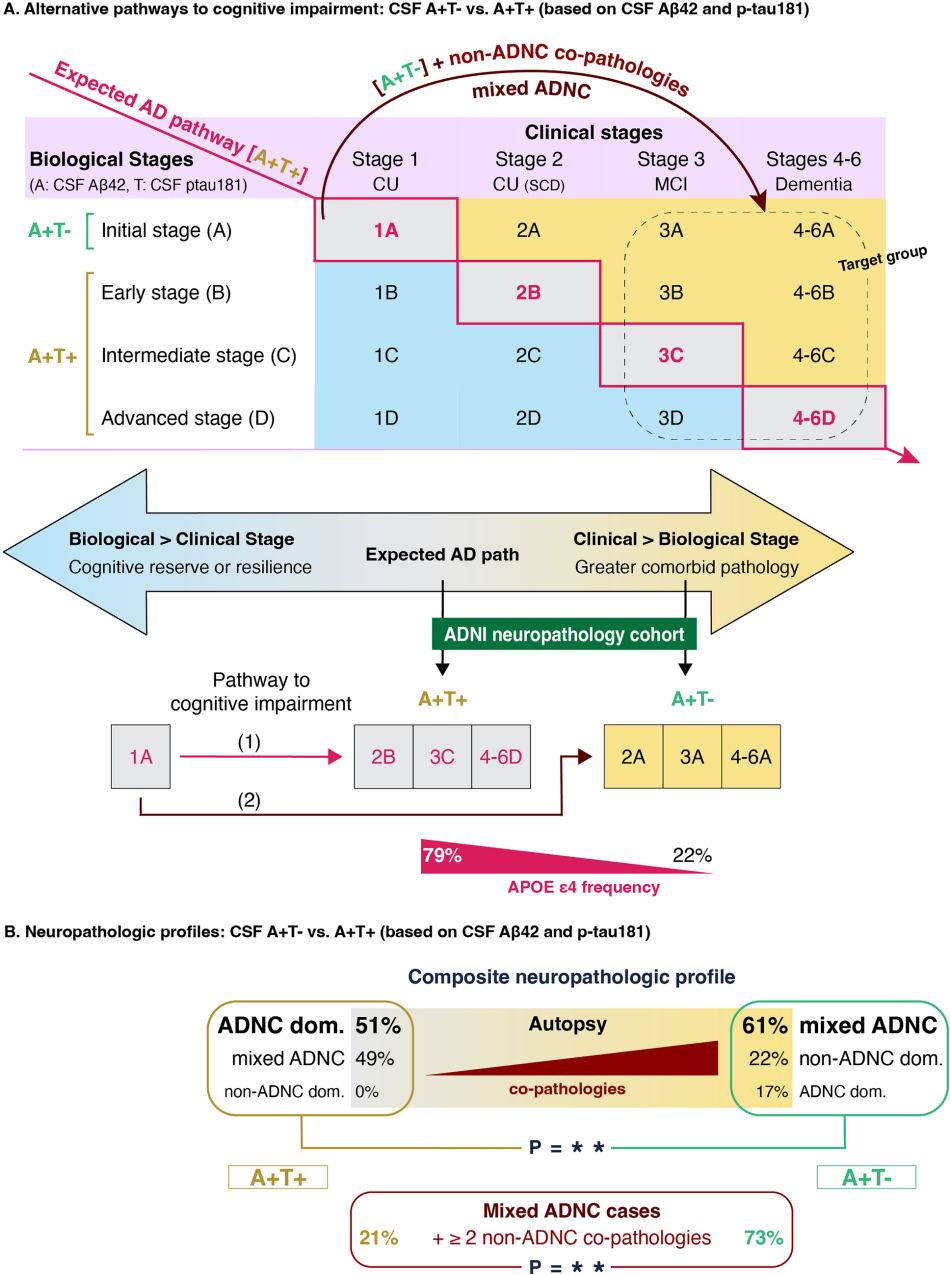
The impact of mixed pathologies on the interpretation of the CSF A/T biomarker system (based on CSF Aβ42 and p-tau181) with respect to the most recent revised AD criteria of the Alzheimer’s Association Workgroup (Jack CR Jr, et al., 2024). The presence of non-ADNC pathologies can influence the clinical interpretation of the CSF A/T biomarker system. **A** Alternative pathways to cognitive impairment. Cognitively impaired CSF A + T + individuals have followed the expected AD pathway (1), but CSF A + T − individuals may have reached severe cognitive impairment through an alternative pathway based on co-pathologies (2). **B** Neuropathologic profiles: CSF A + T − vs A + T + . The composite underlying neuropathology differs significantly between cognitively impaired CSF A + T − and A + T + individuals. Mixed ADNC was defined as the copresence of ADNC and at least one other non-ADNC pathology associated with cognitive impairment. *A − /* + β-amyloid negativity/positivity, *AD* Alzheimer’s disease, *ADNC* Alzheimer’s disease neuropathologic change, *ADNI* Alzheimer’s Disease Neuroimaging Initiative, *CSF* cerebrospinal fluid, *T − /* + tau negativity/positivity

The revised criteria of the Alzheimer’s Association Workgroup for the diagnosis and staging of AD, were proposed to distinguish between the clinical and biological dimensions of the disease by introducing a two-axis framework. The clinical axis represents the severity of cognitive impairment, whereas the biological axis is anchored by Aβ biomarkers and further stratified by tau biomarker levels to stage disease progression. According to this framework, a typical AD trajectory is marked by tau biomarker positivity (biological stage ≥ B) which typically precedes the MCI or dementia stage (clinical stage ≥ 3; Fig. 3A, pathway (1)). In our study, the CSF A + cognitively impaired individuals followed two distinct trajectories. The first aligned with the aforementioned expected diagonal (CSF A + T + ; clinical stage ≥ 3 with biological stage > B). The second deviated from this expected diagonal showing advanced clinical stage despite earlier biological stage (CSF A + T − ; clinical stage ≥ 3 with biological stage < B, Fig. 3A, pathway (2)). Interestingly, the composite neuropathologic profile differed significantly between the CSF A + T − and A + T + groups. Among CSF A + T + individuals, 51% of cases had ADNC intermediate/high without significant co-pathologies associated with cognitive impairment (i.e., ADNC dominant), while 49% of cases had mixed ADNC. In contrast, only 17% of CSF A + T − individuals showed ADNC dominant pathology; the majority (61%) had mixed ADNC, with a significantly greater number of co-pathologies compared to the mixed ADNC cases in the CSF A + T + group. Additionally, 22% of CSF A + T − individuals showed a non-ADNC dominant pathology, showing no or only low ADNC (Fig. 3B). These findings suggest that CSF A + T − individuals can reach considerable cognitive impairment through an alternative trajectory—namely a mixed pathologies pathway. Supporting this interpretation, we found a significantly higher frequency of APOE ε4 carriers—the strongest genetic risk factor for AD [62]—in CSF A + T + individuals compared to CSF A + T − individuals (79% vs. 22%), consistent with a more canonical AD-driven process (Fig. 3). Collectively, our findings, together with growing evidence that cognitive decline often results from multiple pathologies rather than a single dominant one, [53, 59, 65, 71] support the notion that distinct pathways—potentially reflected in CSF A/T biomarker status—could eventually lead to severe cognitive impairment.

The decoupling of CSF Aβ and tau biomarkers in the context of mixed pathologies underscores the potential need to revisit how we interpret clinical trials, select therapeutic targets, and optimize patient selection for disease-modifying therapies. In both currently available FDA-approved therapies—lecanemab and donanemab—subgroup analyses, whether pre-specified or post hoc, consistently showed that individuals with the lowest tau burden derived the greatest clinical benefit [17, 64]. At first glance, this may appear contradictory to our findings; however, the interpretation is more nuanced. Compared to CSF A + T + individuals, CSF A + T − individuals may exhibit essentially indistinguishable clinical phenotypes which are associated with a greater number of co-pathologies but less extensive tau pathology (Table 3).This implies that Aβ positive cognitively impaired individuals with low tau levels, despite the presence of additional non-ADNC pathologies (i.e., mixed ADNC), may still respond better to Aβ-targeting therapy than their Aβ positive counterparts with high tau levels, who exhibit more advanced AD pathology [64]. This notion aligns with the amyloid hypothesis, based on which the greatest opportunity for therapeutic impact is early in the disease process, when AD-related tau pathology is still spatially restricted to the medial temporal lobe and remains dependent on the presence of upstream Aβ pathology. Conversely, Aβ positive cognitively impaired individuals with high tau levels, who harbor more widespread tau pathology (i.e., Braak stages V–VI) that drives neurodegeneration, may benefit more from dual-targeting strategies combining both Aβ and tau immunotherapies. Lastly, in the context of optimizing patient selection for disease-modifying therapies, it is important to further investigate and identify Aβ positive cognitively impaired individuals in whom ADNC may not be sufficient to account for cognitive impairment (e.g., cases with low ADNC). These individuals, who may represent up to 20% of the CSF A + T − group, could require tailored therapeutic strategies.

Our study also yielded two interesting findings beyond our main research questions. The first concerns the pathologic background of the CSF A − T + biomarker profile. Although it has been suggested that the A − T + biomarker profile may represent an age-related neurodegenerative process such as primary age-related tauopathy (PART), these cases have not been strongly associated with specific neuropathologic entities. In line with current literature, [47, 72] the CSF A − T + cases in our study, accounting for approximately 13% of the sample (*n* = 10), constituted a heterogeneous group. These individuals were relatively older than the other groups, most were cognitively impaired (80%), and they exhibited a diverse spectrum of neuropathologies, with PART identified in only a subset of cases (40%). It should also be noted that two CSF A − T + cases were positive for Aβ PET (both > 80 Centiloids), suggesting that a proportion of CSF A − T + cases may represent false CSF Aβ negatives (Supplementary Table 3, Supplementary Fig. 4). However, given the limited number of CSF A − T + cases in our sample, firm conclusions about their clinical and pathological characteristics cannot be drawn, and further research is warranted. The second noteworthy finding concerns the performance of the CSF α-syn biomarker (i.e., synuclein seed amplification assay, Amprion) in detecting LB pathology. CSF α-syn demonstrated good-to-high sensitivity in detecting LB limbic/neocortical (86%) and excellent sensitivity in detecting LB neocortical (100%), but moderate specificity and low positive predictive value in both scenarios (Supplementary Table 2). The high sensitivity of CSF α-syn in detecting LB pathology has also been confirmed by prior studies [67]. The detection of α-synuclein in biofluids represents a promising initial step toward improving the diagnosis of synucleinopathies.

The contributions and strengths of our study stem from the characteristics of our sample and the novelty of our approach. Importantly, the selection of our study sample (i.e., ADNI) allowed us to capture the full spectrum of age-dependent pathologies and include participants exhibiting diverse combinations of ADNC and non-ADNC pathologies that can contribute to cognitive impairment. Our non-hierarchical approach emphasizes the contribution of total pathologic burden. Clinical characterization, longitudinal follow-up, and comprehensive postmortem neuropathologic assessment enabled detailed individual profiling and enhanced the clinical relevance of our findings. Despite its exploratory nature, we believe our study provides proof-of-concept evidence regarding neuropathologic differences between the CSF A + T − and A + T + groups, as well as the influence of co-pathologies in the clinical interpretation of CSF A/T biomarker system. Furthermore, our methodological approach of binarizing neuropathologies—assessing whether the burden of each alone was sufficient to plausibly contribute to cognitive impairment—could be applied in the design of future studies investigating the additive impact of co-pathologies.

The generalization of our findings is constrained by certain limitations. An important limitation of our study is the modest sample size. In combination with the absence of cognitively unimpaired individuals, this may have led to an overestimation of the frequency of co-pathologies or to the identification of false-positive neuropathologic profiles associated with cognitive impairment. While a biomarker-pathology study offers clear advantages in evaluating biomarker profiles, binarizing the extent of these neuropathologies may simplify their association with objective cognitive impairment. Similarly, the definition of ‘ADNC dominant’ is not universally accepted and is based on our approach, which, although thorough, is limited by the aforementioned binarization. We also need to highlight the limitations associated with the performance of the CSF p-tau181 biomarker as a “T” marker (Supplementary Table 2) compared with tau PET. Although we employed a CSF p-tau181 cut-off validated against tau PET within the same cohort, [16] it is important to note that an abnormal CSF p-tau181 level is not synonymous with extra-medial temporal lobe tau pathology, as is the case with tau PET [49]. The association between CSF p-tau181 and the spatial progression of tau pathology appears to be complex, particularly in relation to Aβ burden, although consistent increases in the current sample were associated with Braak stage ≥ V (Supplementary Fig. 3B), and this warrants further investigation [35]. Additionally, the time lapse between the CSF sampling and neuropathologic assessments is a common limiting factor in this type of studies. The advanced age of participants and the underrepresentation of clinical comorbidities in our study, compared to previously reported findings, [23, 55] likely reflect the design of the ADNI cohort and highlight the need for caution when generalizing our findings to younger cohorts and typical memory clinic populations. Future studies should also span both cognitively unimpaired and cognitively impaired individuals, from community-based to memory clinic-based populations; encompass diverse races, ethnicities, and socio-economic strata; and include assessments of additional cognitive domains that may be related to specific pathologies, as well as other biomarker modalities (e.g., plasma) [68].

In conclusion, although CSF A + T − and A + T + cognitively impaired cases may be essentially indistinguishable clinically, their underlying neuropathologic profiles differ. The frequency of the ADNC dominant profile is low in the CSF A + T − group. Furthermore, when mixed ADNC is present, the CSF A + T − group exhibits a greater number of co-pathologies than the CSF A + T + group. Our findings highlight that co-pathologies may have a significant impact on the clinical interpretation of CSF A/T biomarkers. In cases where low CSF Aβ42 and low p-tau181 are detected, there should be a higher clinical suspicion that cognitive impairment may stem from the cumulative effect of mixed pathologies and the overall pathologic burden, rather than being the exclusive outcome of ADNC. Elucidating the contribution of co-pathologies to cognitive impairment could improve the clinical implementation of disease-modifying therapies and bring us one step closer to individualized patient management.

## Supplementary Information

Below is the link to the electronic supplementary material.

Supplementary file1 (DOCX 2631 KB)

## Data Availability

The ADNI data analyzed in the current study are publicly available by the Laboratory of Neuro Imaging (https://ida.loni.usc.edu/login.jsp?project=ADNI).
