# Supplementary material for: Neuropathologic correlates of cognitive impairment in Alzheimer’s disease with discordant CSF biomarker profiles: co-pathologies in focus
Source: Acta Neuropathol. 2025 Nov 27;150(1):57. doi: 10.1007/s00401-025-02960-w (PMC12657578; doi:10.1007/s00401-025-02960-w)
Supplement: Supplementary file 1 — Supplementary file1 (DOCX 2631 KB) [file 401_2025_2960_MOESM1_ESM.docx]

**Supplementary Material**

**Methods**

**1. Neuropathology**

According to [NACC guidelines](https://files.alz.washington.edu/documentation/np11-guidebook.pdf), evidence of AD neuropathologic change (ADNC) was classified as absent, low, intermediate, or high. Intermediate or high ADNC was considered consistent with a neuropathological diagnosis of AD. For this study, the category of primary age-related tauopathy (PART) was reserved for cases with ‘definite’ (absence of Aβ pathology (Thal phase 0) and Braak NFT stages I-IV) or ‘possible’ (modest Aβ pathology (e.g., Thal phases 1 or 2) and Braak NFT stages I-IV) PART. Lewy body (LB) pathology was evaluated based on its regional distribution. Neocortical or limbic distributions of LB pathology were considered a potential contributor to cognitive impairment. TDP-43 pathology was evaluated in standardized regions (Perrin RJ, et al., 2024). In the absence of evidence for frontotemporal lobar degeneration associated with TDP-43 inclusions (FTLD-TDP-43), staging for limbic-predominant age-related TDP-43 encephalopathy neuropathologic changes (LATE-NC) was applied. Because the NACC form (and therefore the ADNI data currently available from LONI) does not readily support the original or updated staging systems for LATE-NC (Nelson PT, et al., 2019, 2023), the classification of cases used in this article was obtained after communication with the ADNI Neuropathology Core (NPC) and a detailed review of cases identified as having TDP-43 pathology. LATE-NC stages 2 – 3 were evaluated as a potential contributing factor to cognitive impairment. Hippocampal sclerosis (HS), reserved for cases with marked neuronal loss in CA1 and subiculum out of proportion to ADNC tauopathy in these areas, was reported dichotomously as present (unilateral or bilateral) or absent, and its presence was considered significant for cognitive impairment. Similarly, standard assessment was performed for non-ADNC tau inclusions, namely FTLD-tau (e.g., progressive supranuclear palsy (PSP), corticobasal degeneration (CBD), Pick’s disease, argyrophilic grain disease (AGD) etc.). Especially for AGD, staging information (not collected by NACC neuropathology form versions 10 and 11) was obtained after communication with the ADNI NPC, and stages II – III were considered as potentially contributing to cognitive impairment. Neuronal loss in substantia nigra was also dichotomously rated as present (for moderate or severe neuronal loss) or absent (for mild or no neuronal loss). Finally, vascular brain injury (VBI) was defined as the presence of old infarcts (including lacunes) or hemorrhages, grossly observed, or old microvascular lesions (MVLs, microinfarcts/microbleeds). Individuals with evidence of chronic traumatic brain injury, central nervous system infection, and primary brain tumors were excluded in the absence of a detailed medical history related to these conditions.

**2. Longitudinal cognitive modelling**

We used separate linear mixed-effects models for MMSE and ADNI-Mem to evaluate longitudinal cognitive performance. To compare the rate of cognitive decline across the CSF A/T groups, we included an interaction term for time from biomarker testing by CSF A/T group. Random slope and intercept were implemented in the model for accounting the heterogeneity in cognitive trajectories across individuals. The best model was selected using Akaike information criterion.

**Supplementary Tables**

**Supplementary Table 1**. ADNI files and R packages

| **ADNI files that were used in our analysis** | | |
| --- | --- | --- |
| **Category** | **What we used** | **Name of the ADNI file** |
| **Demographics** | Age, sex, education | PTDEMOG.csv |
| **Clinical data** | Clinical diagnosis | DXSUM_PDXCONV_ADNIALL.csv |
|  | Clinical visits | REGISTRY.csv |
| **Medical history** | Medication | RECCMEDS.csv |
|  | Clinical history | RECMHIST.csv |
|  | ADNI3 initial assessment | INITHEALTH.csv |
| **APOE genotype** | Presence of APOE4 genes | APOERES.csv |
| **Neuropsychological data** | MMSE | MMSE.csv |
|  | ADNI composite memory | UWNPSYCHSUM_01_23_23.csv |
| **Neuropathological data** | The whole burden of pathology based on NACC guidelines | NEUROPATH.csv (downloaded on June 26, 2024) |
| **CSF biomarkers** | CSF Aβ1-42 | UPENNBIOMK_MASTER_FINAL.csv |
|  | CSF ptau181 |  |
|  | CSF a-syn | AMPRION_ASYN_SAA.csv (version September 2023) |
| **PET imaging** | β-amyloid PET | All available β-amyloid PET scans of the ADNI participants used in our study were downloaded through the Image and Data Archive (IDA) |
|  | AV45  FBB  PiB |  |
| **Software used in analysis** |  |  |
|  | **What we used** | **Name of the package/Information** |
| R version 4.1.1 (2021-08-10)  RStudio Version 2023.6.0.421 | Linear mixed effects models | lme4 |
|  | Linear models | stats, emmeans |
|  | Venn diagrams | nVennR |
| MATLAB R2024a | Analysis and quantification of β-amyloid PET imaging | Standard procedures based on GAAIN project  (<https://www.gaain.org>) |
| SPM12 |  |  |

**Supplementary Table 2.** The performance of CSF Aβ42 and p-tau181 in detecting AD pathologic features, and CSF α-syn in detecting LB pathology.

| **Biomarker**  **performance** | **CSF A+ (Aβ42 ≤ 981 pg/mL) ^1^** | | | **CSF T+ (p-tau181 ≥ 24.3 pg/mL) ^1^** | | | | **CSF α-syn+ ^2^** | |
| --- | --- | --- | --- | --- | --- | --- | --- | --- | --- |
| **Measures** | Thal phase ≥ 2 | CERAD **^3^** | ADNC **^4^** | Braak NFT  stage ≥ III | Braak NFT  stage ≥ V | CERAD **^3^** | ADNC **^4^** | LB **^5^**  lim/neο | LB **^6^**  neo |
| Se | 84% | 91% | 88% | 72% | 83% | 78% | 72% | 86% | 100% |
| Sp | 90% | 65% | 76% | 69% | 76% | 70% | 65% | 79% | 76% |
| PPV | 98% | 86% | 93% | 90% | 88% | 86% | 88% | 63% | 53% |
| NPV | 45% | 75% | 65% | 39% | 68% | 57% | 39% | 93% | 100% |

**^1^** CSF Aβ42 and CSF p-tau181 levels were measured with the fully automated Roche Elecsys immunoassay. **^2^** CSF α-syn positivity was determined using the synuclein seed amplification assay at the Amprion Clinical Laboratory. **^3^** CERAD score moderate/frequent. **^4^** ADNC intermediate/high. **^5^** LB limbic/neocortical. **^6^** LB neocortical. [AD = Alzheimer’s disease, ADNC = Alzheimer’s disease neuropathologic change, CERAD = consortium to establish a registry for Alzheimer’s disease, LB = Lewy body pathology, NFT = neurofibrillary tangles, NPV = negative predictive value, PPV = positive predictive value, Se = sensitivity, Sp = specificity]

**Supplementary Table 3.** Individuals with discrepancy between CSF Aβ42 status and Aβ PET status.

| **Biomarker status** | | | | **Clinical diagnosis** | |  | **Autopsy** | |
| --- | --- | --- | --- | --- | --- | --- | --- | --- |
| **CSF ^1^** | **CSF Aβ42** (pg/mL) | **Aβ PET** (CL) | **PET – CSF** | **At CSF** | **Last assessment** | **CSF – Death** | **ADNC** | **Other pathologies** |
| ***1^st^ individual*** *(67 y)* **^2^** | | | | | | | | |
| A-T- | 1291.0 pg/mL | A+ (33.4 CL) | < 1 year | CU | CU | 3.3 years | Low | – |
| ***2^nd^ individual* ^3^** *(85 y)* | | | | | | | | |
| A-T+ | 1082.0 pg/mL | A+ (94.7 CL) | < 1 year | MCI | MCI | 4.8 years | High | – |
| ***3^rd^ individual*** *(80 y)* | | | | | | | | |
| A-T+ | 998.2 pg/mL | A+ (84.0 CL) | < 1 year | Dementia | Dementia | 6.2 years | High | AGD stage I,  LB neocortical,  TDP-43 (amygdala), VBI |
| ***4^th^ individual*** *(88 y)* | | | | | | | | |
| A+T- | 1. pg/mL | A- (9.5 CL) | < 1 year | Dementia | Dementia | < 1 year | Intermediate | AGD stage II,  LATE stage 2, HS |

**^1^** The CSF sample with the minimal time difference from the autopsy was considered the a priori time point for biomarker testing to define the individual's biomarker status. **^2^** The age of the individual at the time of CSF sampling. **^3^** The 2^nd^ individual is the only of these four individuals that had another CSF sampling. The other CSF sample was taken 6.84 years before death; CSF status = A+T+ with Aβ42 levels = 963.2 pg/mL. [A = β-amyloid, ADNC = Alzheimer disease neuropathologic change, AGD = argyrophilic grain disease, CL = centiloids, CSF = cerebrospinal fluid, CU = cognitively unimpaired, LATE-NC = limbic-predominant age-related TDP-43 encephalopathy - neuropathologic change, HS = hippocampal sclerosis, LB = Lewy bodies, MCI = mild cognitive impairment], PET = positron emission tomography, T = tau, VBI = vascular brain injury]

**Supplementary Table 4.** Clinical comorbidities across the CSF A/T groups

| **Medical history** | **A-T-, N = 10 ^1^** | **A-T+, N = 10 ^1^** | **A+T-, N = 18 ^1^** | **A+T+, N = 39 ^1^** | **p-value ^2^** | |
| --- | --- | --- | --- | --- | --- | --- |
|  |  |  |  |  | **All AT/ groups** | **A+T- vs A+T+** |
| **Cerebrovascular disease risk factors** |  |  |  |  |  |  |
| Smoking | 8 / 10 (80%) | 3 / 10 (30%) | 7 / 18 (39%) | 12 / 39 (31%) | **0.044** | NS |
| Hypertension/Antihypertensive medication | 9 / 10 (90%) | 7 / 10 (70%) | 6 / 18 (33%) | 28 / 39 (72%) | **0.011** | **0.009** |
| Diabetes mellitus/Antidiabetic medication | 1 / 10 (10%) | 1 / 10 (10%) | 2 / 18 (11%) | 4 / 39 (10%) | > 0.9 |  |
| Hyperlipidemia/Antihyperlipidemic medication | 6 / 10 (60%) | 9 / 10 (90%) | 11 / 18 (61%) | 31 / 39 (79%) | 0.2 |  |
| History of cardiovascular disease | 6 / 10 (60%) | 3 / 10 (30%) | 2 / 18 (11%) | 14 / 39 (36%) | 0.052 |  |
| Atrial fibrillation/flutter | 0 / 10 (0%) | 0 / 10 (0%) | 0 / 18 (0%) | 1 / 39 (2.6%) | > 0.9 |  |
| Left ventricular hypertrophy | 0 / 10 (0%) | 0 / 10 (0%) | 0 / 18 (0%) | 0 / 39 (0%) |  |  |
| Stroke/TIA | 0 / 10 (0%) | 1 / 10 (10%) | 2 / 18 (11%) | 4 / 39 (10%) | 0.9 |  |
| VRF+ (VRF > 1) ^3^ | 8 / 10 (80%) | 8 / 10 (80%) | 11 / 18 (61%) | 29 / 39 (74%) | 0.7 |  |
| **Psychiatric comorbidities** |  |  |  |  |  |  |
| Depression/Antidepressant medication | 3 / 10 (30%) | 2 / 10 (20%) | 11 / 18 (61%) | 23 / 39 (59%) | 0.071 |  |
| Psychosis/Antipsychotic medication | 1 / 10 (10%) | 0 / 10 (0%) | 4 / 18 (22%) | 6 / 39 (15%) | 0.5 |  |
| Sedatives | 1 / 10 (10%) | 0 / 10 (0%) | 0 / 18 (0%) | 6 / 39 (15%) | 0.2 |  |
| **Other** |  |  |  |  |  |  |
| Movement disorders ^4^ | 0 / 10 (0%) | 1 / 10 (10%) | 3 / 18 (17%) | 4 / 39 (10%) | 0.8 |  |
| Medication for AD | 3 / 10 (30%) | 6 / 10 (60%) | 16 / 18 (89%) | 37 / 39 (95%) | **< 0.001** | NS |
| ACB+ (ACB > 3) ^5^ | 7 / 10 (70%) | 1 / 10 (10%) | 6 / 18 (33%) | 19 / 39 (49%) | **0.034** | NS |

^1^ Median (IQR); n / N (%). ^2^ Fisher's exact test; Kruskal-Wallis rank sum test. ^3^ The presence of VRF burden (VRF+) was defined as the coexistence of two or more of the following conditions: (i) cardiovascular disease, (ii) hypertension (positive medical history or use of antihypertensive medication), (iii) diabetes mellitus (positive medical history or use of antidiabetic medication), (iv) hyperlipidemia (positive medical history or use of antihyperlipidemic medication), (v) stroke or TIA, (vi) smoking (ever or never), (vii) atrial fibrillation, and (viii) left ventricular hypertrophy (Ferrari-Souza JP, et al., 2024). ^4^ The category of Movement disorders includes individuals diagnosed with essential tremor, periodic limb movement disorder, restless leg syndrome, and Parkinson’s disease. ^5^ The ACB score was evaluated using the [ACB calculator](https://www.acbcalc.com/). [A-/+ = β-amyloid negativity/positivity, ACB = anticholinergic cognitive burden, AD = Alzheimer’s disease, IQR = interquartile range, T-/+ = tau negativity/positivity, TIA = transient ischemic attack, VRF = vascular risk factors]

**Supplementary Table 5.** Likelihood that the pathologic findings are associated with a typical, dementia with Lewy bodies, clinical syndrome.^1^

| **Pathologies** | **Alzheimer disease neuropathological change (ADNC)** ^2^ | | |
| --- | --- | --- | --- |
|  | **NIA-AA none/low** | **NIA-AA intermediate** | **NIA-AA high** |
| **Lewy-related pathology** ^2^ |  |  |  |
| Diffuse (neocortical) | 4 ^SN(3)^ | 1 ^SN(1)^ | 11* ^SN(5)^ |
| Limbic (transitional) | 1 | 0 | 5* |
| Brainstem predominant | 2 | 0 | 1 |
| Amygdala predominant | 0 | 1 | 10 |
| Olfactory bulb only | 1 | 0 | 1 |

SN = substantia nigra ^3^

| Likelihood | Low | Intermediate | High |
| --- | --- | --- | --- |

^1^ Modified table based on McKeith IG, et al., 2017. Diagnosis and management of dementia with Lewy bodies. Fourth consensus report of the DLB Consortium. ^2^ ADNC and Lewy-related pathology were assessed based on the [National Institute on Aging-Alzheimer’s Association (NIA-AA) guidelines](https://naccdata.org/data-collection/forms-documentation/np-11). ^3^ SN(number) signifies the number of individuals with moderate of severe neuronal loss in the SN. Two individuals (*) had no available data regarding the evidence of neuronal loss in the SN and two individuals (not presented here; one with high ADNC and one with intermediate ADNC) showed evidence of neuronal loss in the SN but without evidence of other Lewy-related pathology.

**Supplementary Table 6.** Composite neuropathologic profile including VBI in the non-ADNC pathologies across the CSF A/T groups.

| **Pathology** | **A-T- (N = 10)^1^** | **A-T+ (N = 10)^1^** | **A+T- (N = 18)^1^** | **A+T+ (N = 39)^1^** | **p-value^2^** | |
| --- | --- | --- | --- | --- | --- | --- |
|  |  |  |  |  | **All A/T groups** | **A+T- vs A+T+** |
| **Composite neuropathologic profile** | |  |  |  | **< 0.001** | **0.003** |
| ADNC dominant | 2 / 10 (20%) | 2 / 10 (20%) | 2 / 18 (11%) | 14 / 39 (36%) |  |  |
| Mixed ADNC**^3,4^** | 1 / 10 (10%) | 2 / 10 (20%) | 12 / 18 (67%) | 25 / 39 (64%) |  |  |
| Non-ADNC dominant**^5^** | 6 / 10 (60%)**^6^** | 6 / 10 (60%) | 4 / 18 (22%) | 0 / 39 (0%) |  |  |

**^1^** n / N (%). **^2^** Fisher's exact test. **^3^** Mixed ADNC = ADNC intermediate/high + ≥ 1 non-ADNC pathologies that, in isolation, have been associated with cognitive impairment, or VBI. ^4^ PART is not included in the definition of mixed ADNC by default. **^5^** Presence of PART, VBI or any of the non-ADNC pathologies in the absence of ADNC (intermediate/high). **^6^** One A-T- individual (10%) had neither ADNC nor any of the non-ADNC pathologies assessed in this study, nor evidence of VBI. [A-/+ = β-amyloid negativity/positivity, ADNC = AD neuropathologic change, PART = primary age-related tauopathy, T-/+ = tau negativity/positivity, VBI = vascular brain injury]

**Supplementary Figures**

**Supplementary Figure 1**


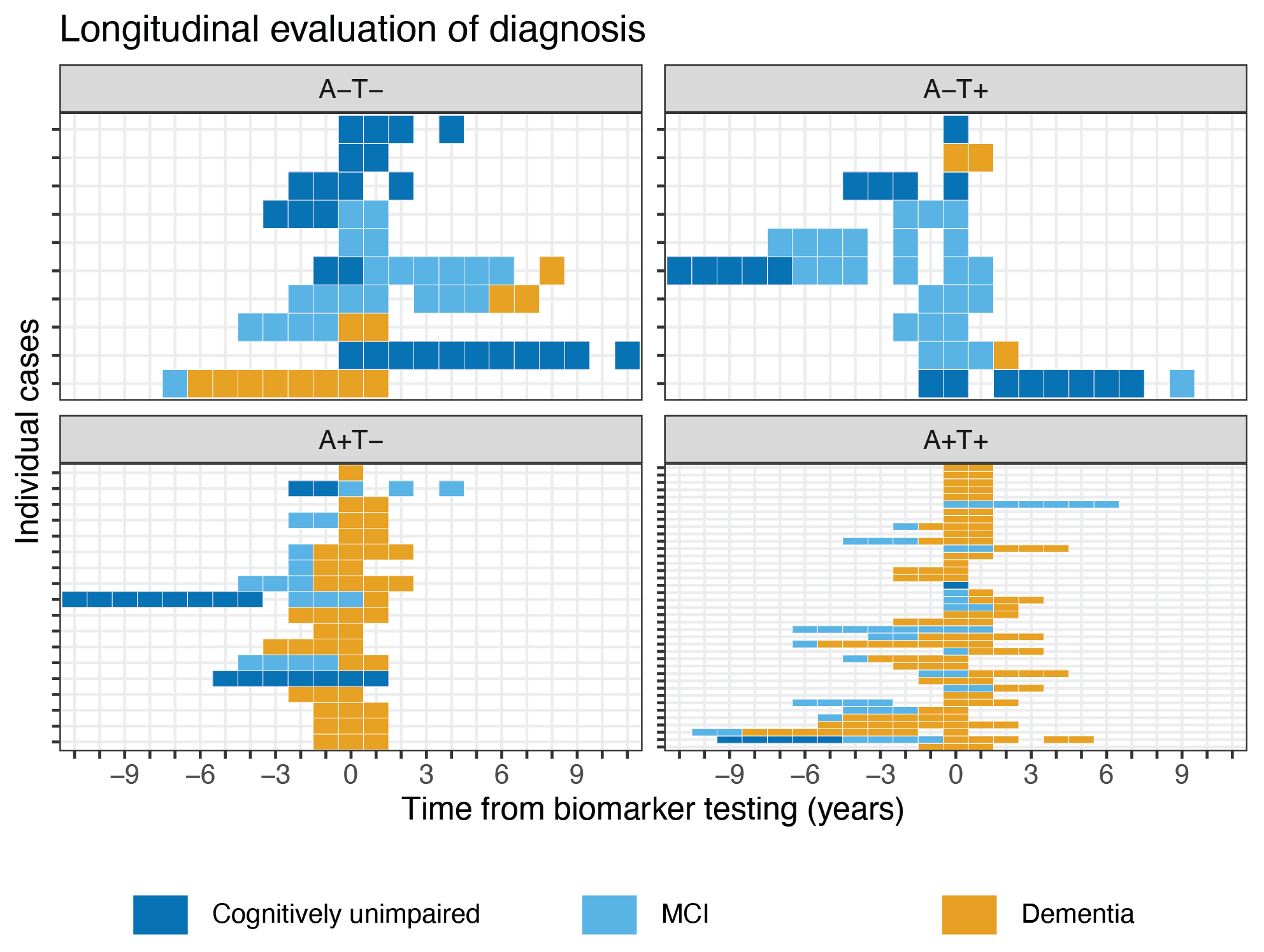


**Supplementary Figure 1**. Longitudinal changes in clinical diagnosis by CSF A/T group and individual. Individuals are longitudinally characterized at each time point based on their clinical diagnosis as cognitively unimpaired, mildly cognitively impaired, or having dementia. The zero time point represents the last ante-mortem CSF sample provided by each participant, which was used to define their CSF A/T biomarker profile. [A-/+ = β-amyloid negativity/positivity, CSF = cerebrospinal fluid, CU = cognitively unimpaired, MCI = mild cognitive impairment, T-/+ = tau negativity/positivity]

**Supplementary Figure 2**

**
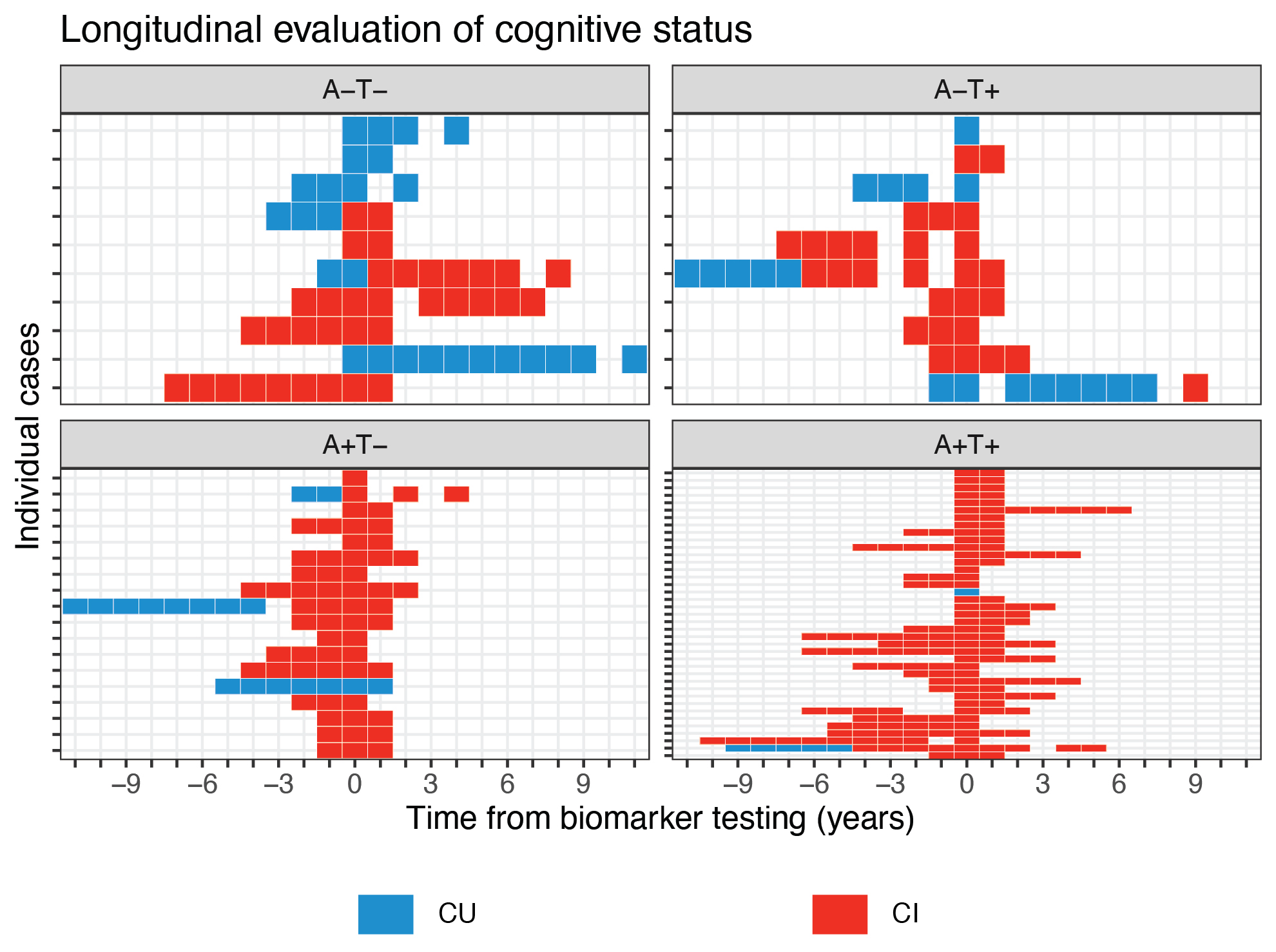
**

**Supplementary Figure 2**. Longitudinal changes in cognitive status by CSF A/T group and individual. Individuals are longitudinally characterized at each time point based on their cognitive status as either cognitively unimpaired or cognitively impaired (mild cognitive impairment or dementia). The zero time point represents the last ante-mortem CSF sample provided by each participant, which was used to define their CSF A/T biomarker profile. [A-/+ = β-amyloid negativity/positivity, CI = cognitively impaired, CSF = cerebrospinal fluid, CU = cognitively unimpaired, T-/+ = tau negativity/positivity]

**Supplementary Figure 3**

**
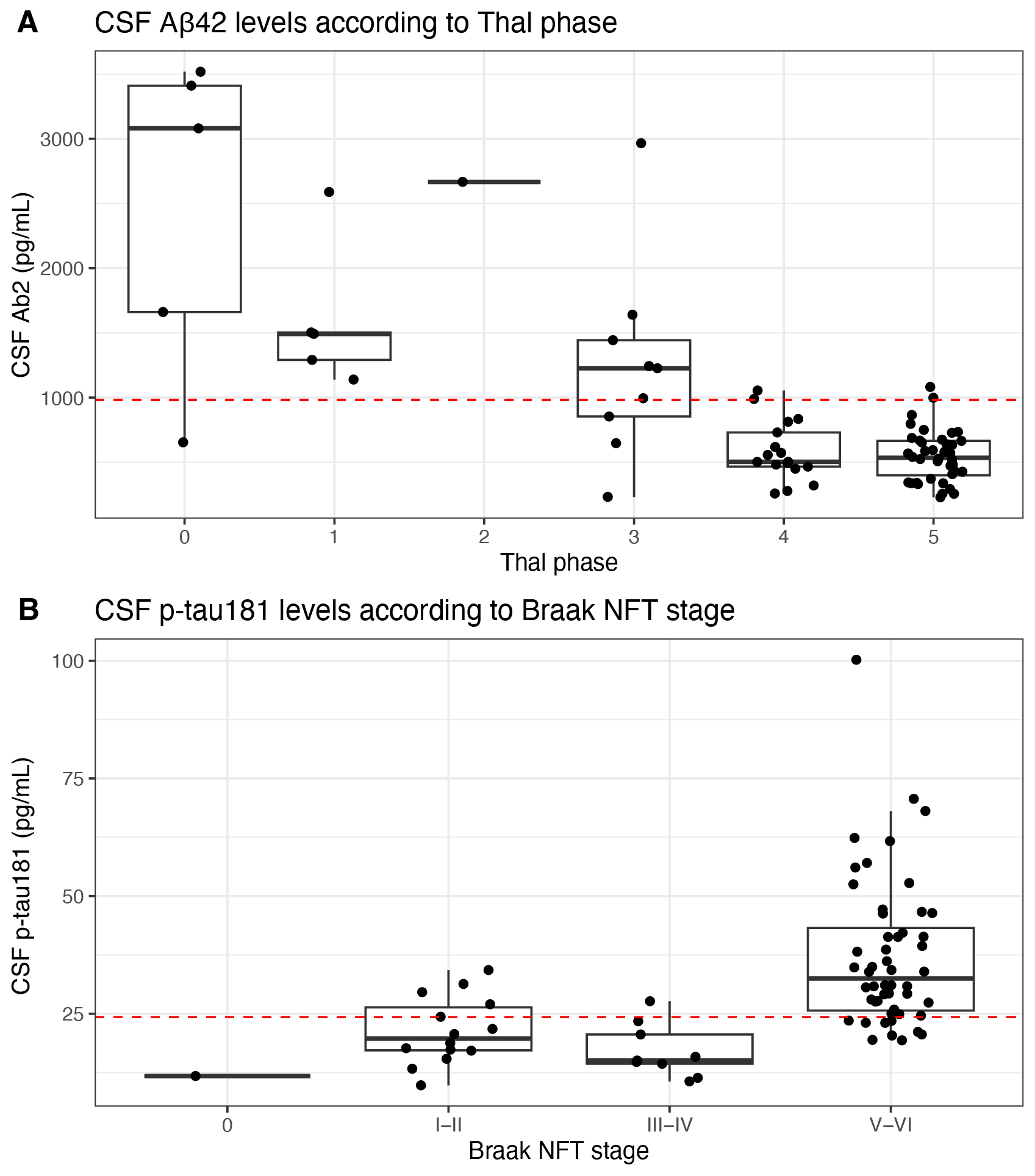
**

**Supplementary Figure 3.** The association of CSF Aβ42 and p-tau181 biomarkers with AD pathologic features. **A**. CSF Aβ42 levels according to Thal phase. The red line indicates the cut-off (Aβ42 ≤ 981 pg/mL) used to define β-amyloid positivity (CSF A+) in our study. **B**. CSF p-tau181 levels according to Braak NFT stage. The red line indicates the cut-off (p-tau181 ≥ 24.3 pg/mL) used to define tau positivity (CSF T+) in our study. One individual classified as CSF T-, with Braak NFT stage I – II, is not shown in this graph due to a CSF p-tau181 level < 8 pg/mL.

**Supplementary Figure 4**

**
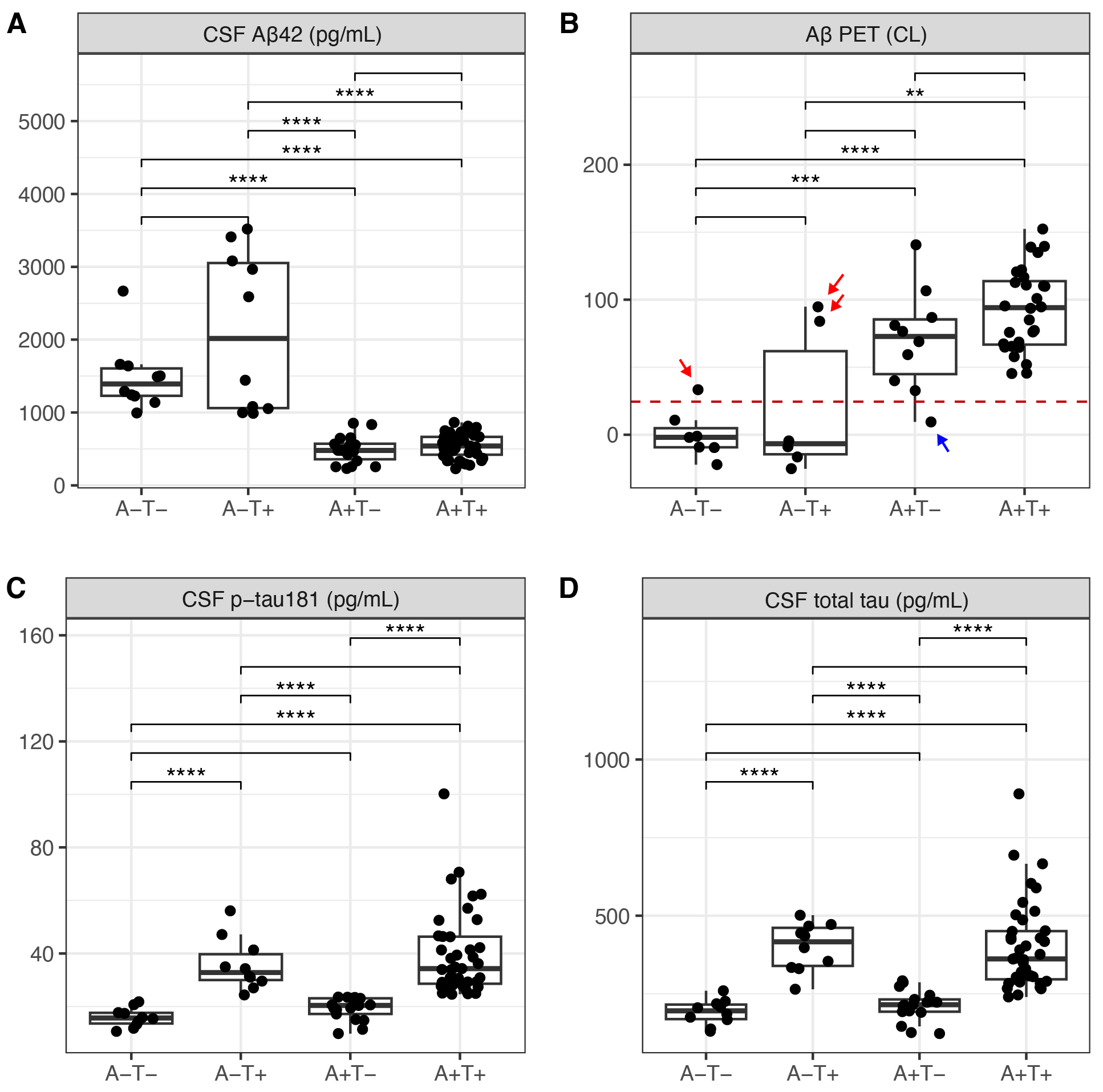
**

**Supplementary Figure 4.** Biomarker levels and comparisons across the CSF A/T groups. **A**. CSF Aβ42 levels across the CSF A/T groups. **B**. Aβ burden measured in centiloids across the CSF A/T groups. The dark red dashed line represents the cut-off of 24.4 CL for defining Aβ PET positivity; the red arrows indicate the individuals who were A- in CSF but A+ in PET, while the blue arrow indicates the individual who was A+ in CSF but A- in PET. More information about these individuals can be found in Supplementary Table 3. **C**. CSF p-tau181 levels across the CSF A/T groups. **D**. CSF total tau levels across the CSF A/T groups. [A-/+ = β-amyloid negativity/positivity, CL = centiloids, CSF = cerebrospinal fluid, PET = positron emission tomography, T-/+ = tau negativity/positivity]

**Supplementary Figure 5**

**
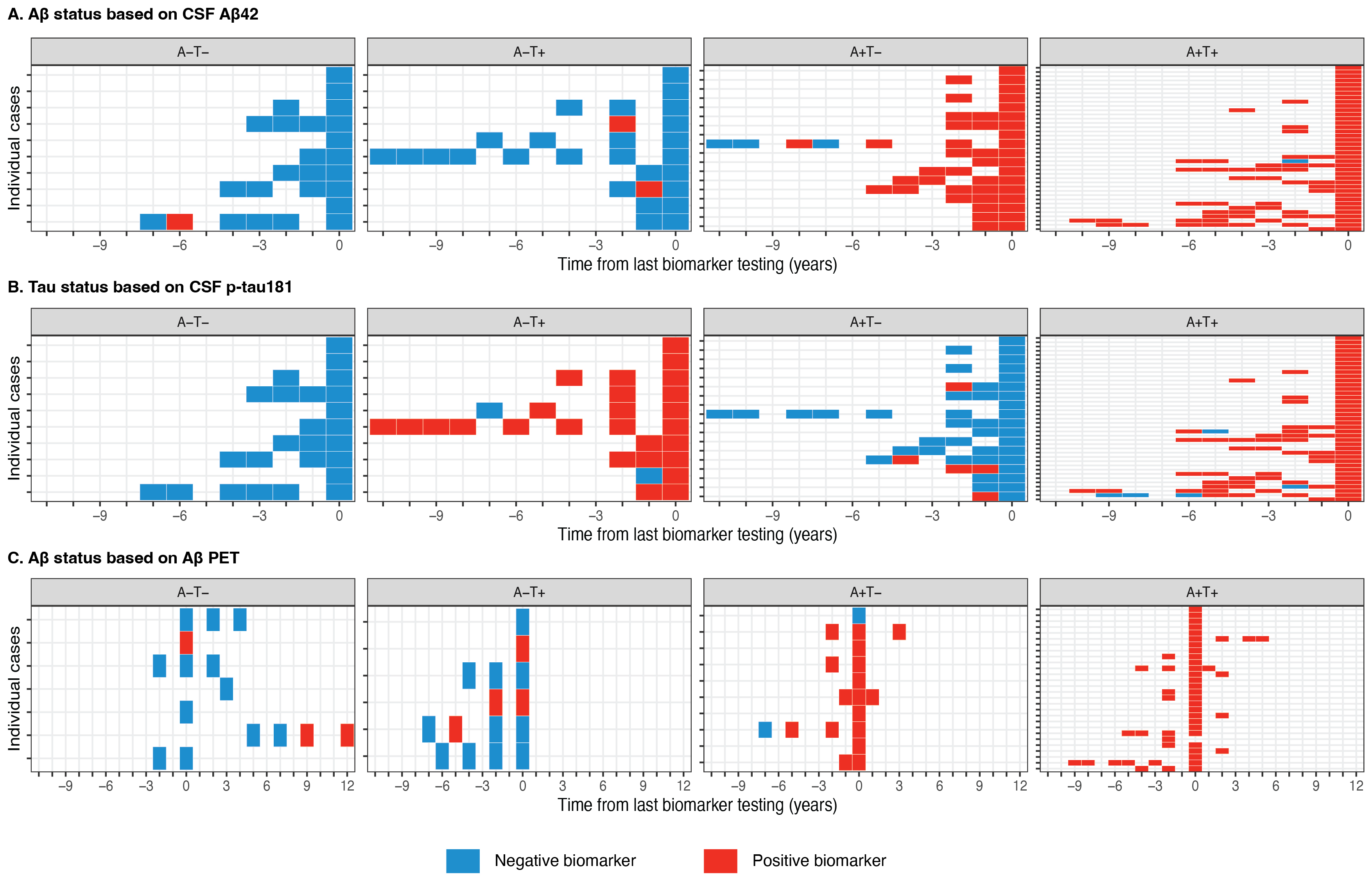
**

**Supplementary Figure 5.** Longitudinal changes in biomarker status by CSF A/T group and individual. **A**. Longitudinal changes in CSF Aβ1-42. **B**. Longitudinal changes in CSF ptau181. **C**. Longitudinal changes in Aβ PET status. The zero time point represents the last ante-mortem CSF sample provided by each participant, which was used to define their CSF A/T biomarker profile. [A-/+ = β-amyloid negativity/positivity, CSF = cerebrospinal fluid, PET = positron emission tomography, T-/+ = tau negativity/positivity]
